# Supplementary material for: Systemic biological mechanisms underpin poor post-discharge growth among severely wasted children with HIV
Source: Nat Commun. 2024 Nov 27;15:10299. doi: 10.1038/s41467-024-54717-w (PMC11603168; doi:10.1038/s41467-024-54717-w)
Supplement: Supplementary file 1 — Supplementary Information [file 41467_2024_54717_MOESM1_ESM.pdf]

Systemic biological mechanisms underpin poor post-discharge growth among severely wasted children with HIV

Supplementary Results

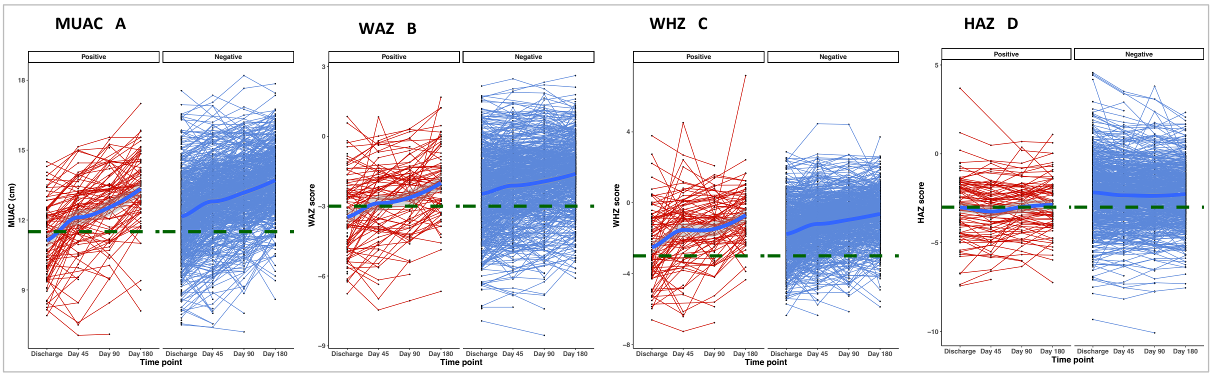

Supplementary Figure 1. Six months growth trajectories for every child by HIV status. (A-D). Line graphs illustrating post-discharge growth trajectories for each child by MUAC, WAZ, WHZ and HAZ respectively. The graphs are fitted with a LOESS function represented by a thick blue line, indicating the overall growth rates by HIV status. Red and blue colours denote children with and without HIV, respectively. Horizontal dotted green lines indicate the cutoffs for severe wasting, underweight and stunting. Abbrevaitions: MUAC, mid-upper arm circumference, WAZ, weight-for-age z score; WHZ, weight-for-height z score and HAZ, height-for-age z score.

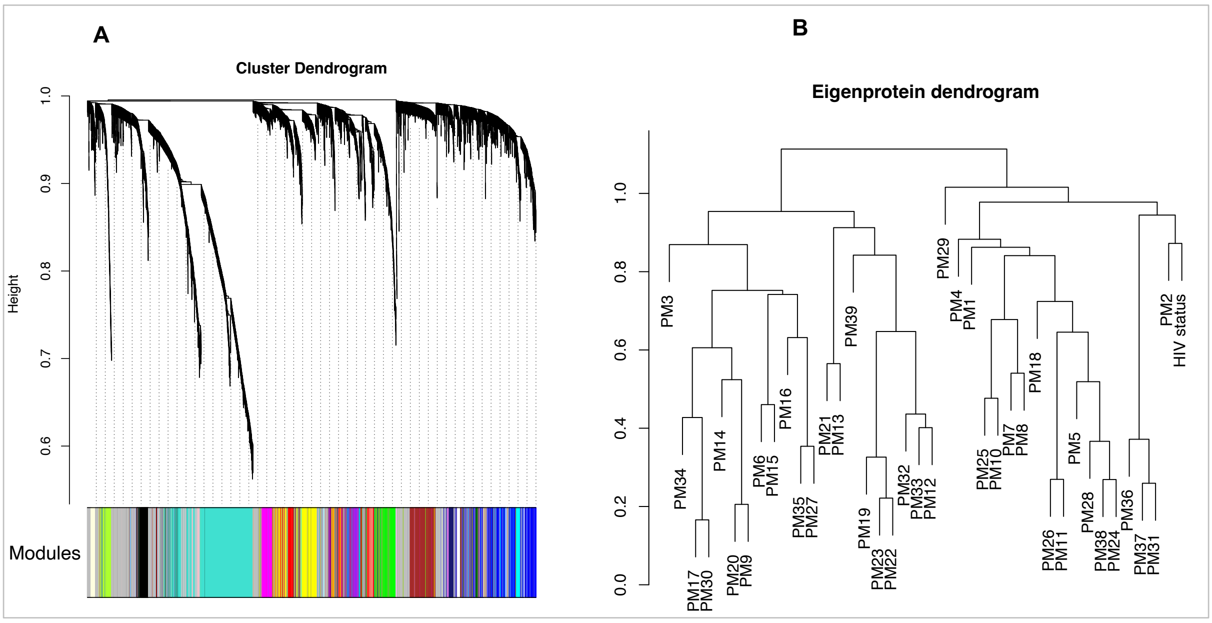

Supplementary Figure 2. Dendrogram of proteins generated from the weighted correlation network analysis. (A). Hierarchical clustering dendrogram of plasma proteome. Forty protein modules (represented by the different colors) were generated from the 7335 plasma proteins. Each branch of the dendrogram represents a single protein, and the coloured bars below the branches designate the corresponding module a protein was assigned to. The dendrogram height is the distance between proteins. (B). Hierarchical clustering dendrogram of the eigenproteins showing relationship between HIV and modules, and also how modules relate with each other.

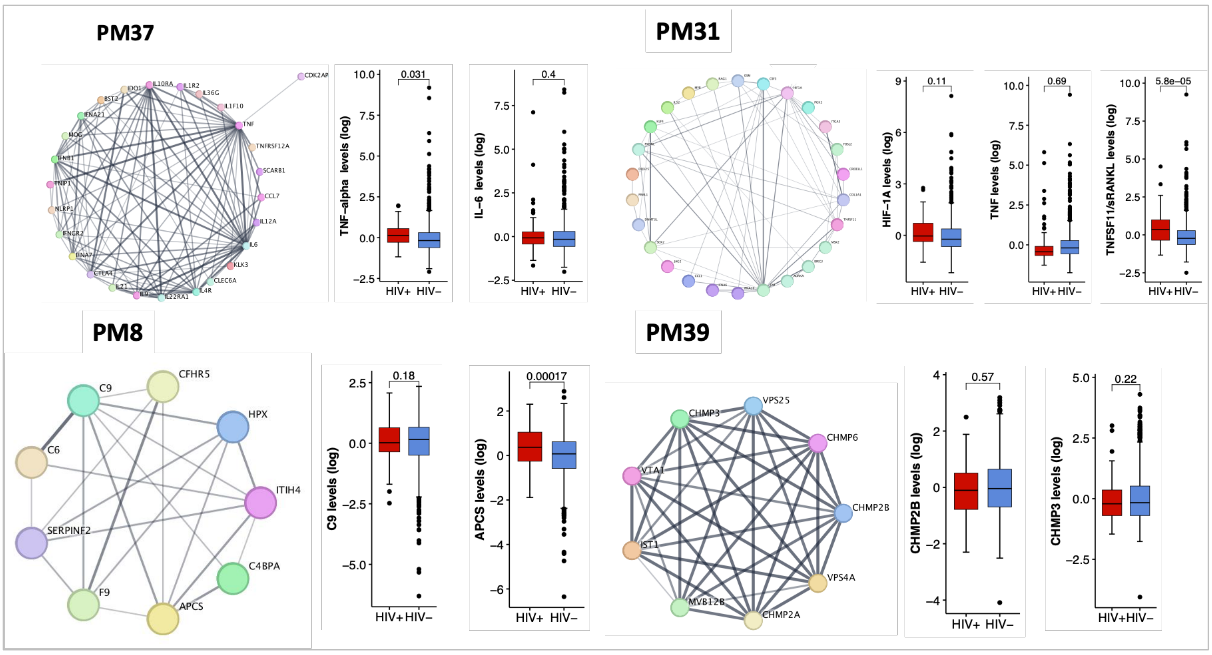

Supplementary Figure 3. Core proteins within modules positively associated with HIV status. Protein connectivity within modules positively associated with HIV. Nodes represent the proteins making up the network. The lines represent edges connecting proteins with dark coloured edges indicating highly correlated proteins in the network. Nodes with high connectivity (many edges) are considered core proteins, controlling the network connection. Box plots show the expression levels of core proteins by HIV status (children with HIV, n = 79; children without HIV, n = 610). A two-sided t-test was used to compare expression levels of core proteins between children with and without HIV, denoted by red and blue colours, respectively. P-values were adjusted for multiple testing using the Bonferroni correction criterion where p-value < 0.05 indicate signficant difference in the expression levels of the core proteins between the two groups. Box plots indicate; median (middle line); 25th (first quartile, Q1) and 75th (third quartile, Q3) percentile (box limits); error bars (whiskers) represent 1.5 multiplied by Q1 and Q3 while single points outside the error bars represent outliers. Abbrevaition: PM, protein module

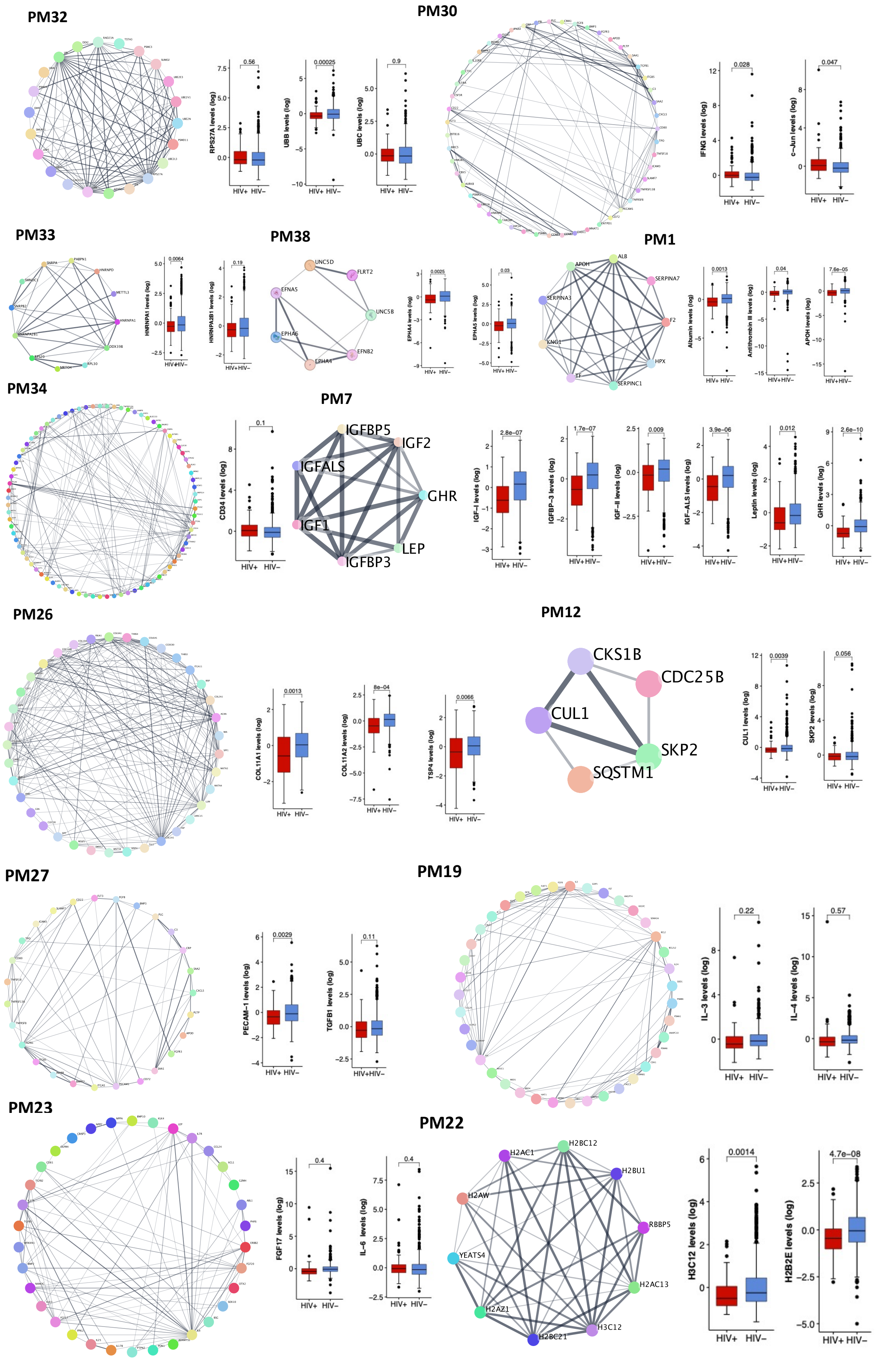
PM32
PM30
PM33
PM38
PM1
PM34
PM7
PM26
PM12
PM27
PM19
PM23
PM22
IGFBP5
IGF2
IGFALS
GHR
IGF1
LEP
IGFBP3
CKS1B
CDC25B
CUL1
SKP2
SQSTM1
H2AC1
H2BC12
H2BU1
H2AW
RBBP5
YEATS4
H2AC13
H2AZ1
H3C12
H2BC21
0.56
0.00025
0.9
0.028
0.047
0.0064
0.19
0.0025
0.03
0.0013
0.04
7.6e−05
0.1
2.8e−07
1.7e−07
0.009
3.9e−06
0.012
2.6e−10
0.0013
8e−04
0.0066
0.0039
0.056
0.0029
0.11
0.22
0.57
0.4
0.4
0.0014
4.7e−08
HIV+ HIV−

Supplementary Figure 4. Core proteins within modules negatively associated with HIV status.

Protein connectivity within modules negatively associated with HIV. Nodes represent the proteins making up the network. The lines represent edges connecting proteins with dark coloured edges indicating highly correlated proteins in the network. Nodes with high connectivity (many edges) are considered core proteins, controlling the network connection. Box plots show the expression levels of core proteins by HIV status (children with HIV, n = 79; children without HIV, n = 610). A two-sided t-test was used to compare expression levels of core proteins between children with and without HIV, denoted by red and blue colours, respectively. P-values were adjusted for multiple testing using the Bonferroni correction criterion where p-value < 0.05 indicate signficant difference in the expression levels of the core proteins between the two groups. Box plots indicate; median (middle line); 25th (first quartile, Q1) and 75th (third quartile, Q3) percentile (box limits); error bars (whiskers) represent 1.5 multiplied by Q1 and Q3 while single points outside the error bars represent outliers. Abbrevaition: PM, protein module

Supplementary Table 1. Characteristics of participants included in the association of HIV status and protein module analysis

**Participant characteristics at discharge**		**HIV^- (n=610)**	**HIV^+ (n=79)**	**Total (N=689)**
Demographic				
Males (%)		345 (57%)	44 (56%)	389 (56%)
Age, months - Median (IQR)		12.8 (7.9 - 17.6)	12.6 (5.7 - 18.0)	12.8 (7.8 - 17.6)
Site – N (%)				
Banfora		115 (19%)	4 (5.1%)	119 (17%)
Blantyre		53 (8.7%)	21 (27%)	74 (11%)
Kampala		176 (29%)	20 (25%)	196 (28%)
Kilifi		78 (13%)	12 (15%)	90 (13%)
Migori		122 (20%)	18 (23%)	140 (20%)
Nairobi		66 (11%)	4 (5.1%)	70 (10%)
Nutritional status at discharge – N (%)				
No wasting		240 (39%)	16 (20%)	256 (37%)
Moderate wasting		144 (24%)	12 (15%)	156 (23%)
Severe wasting		226 (37%)	51 (65%)	277 (40%)
Anthropometry – N (%)				
MUAC (cm)	Median (IQR)<br>Mean (SD)	12.10 (11.10 - 13.20)<br>12.10 (±1.63)	10.95 (9.80 - 12.30)<br>11.00 (±1.59)	12.05 (10.90 - 13.05)<br>12.00 (±1.66)
WAZ score	Median (IQR)<br>Mean (SD)	-2.43 (-3.67 to -1.29)<br>-2.52 (±1.75)	-3.72 (-4.80 to -2.35)<br>-3.55 (±1.63)	-2.60 (-3.86 to -1.38)<br>-2.64 (±1.77)
WHZ score	Median (IQR)<br>Mean (SD)	-1.84 (-2.89 to -0.70)<br>-1.80 (±1.60)	-2.74 (-4.04 to -1.14)<br>-2.58 (±2.06)	-1.90 (-2.97 to -0.78)<br>-1.89 (±1.67)
HAZ score	Median (IQR)<br>Mean (SD)	-2.13 (-3.38 to -1.09)<br>-2.22 (±1.87)	-3.02 (-4.24 to -1.86)<br>-3.08 (±1.65)	-2.24 (-3.51 to -1.15)<br>-2.32 (±1.87)
Oedema - Yes		16 (2.6%)	3 (3.8%)	19 (2.8%)
Clinical illness at admission – N (%)				
Diarrhoea		244 (40%)	35 (44%)	279 (40%)
Pneumonia		292 (48%)	36 (46%)	328 (48%)
Malaria Positive (RDT)		128 (21%)	5 (6.3%)	133 (19%)
Measles		14 (2.3%)	1 (1.3%)	15 (2.2%)
Sepsis		26 (4.3%)	6 (7.6%)	32 (4.6%)
Respiratory Pulmonary TB		12 (2.0%)	8 (10%)	20 (2.9%)
On Co-trimoxazole prophylaxis and Antiretroviral treatment at admission – N (%)				
Cotrimoxazole prophylaxis		4 (0.7%)	20 (25%)	24 (3.5%)
Antiretroviral treatment		–	16 (20%)	–
Data are median (IQR), mean (SD) or count, n (%). RDT – rapid diagnostic test; TB – tuberculosis; MUAC – mid-upper arm circumference; WAZ – weight-for-age; WHZ – weight-for-height; HAZ – height-for-age; IQR – interquartile range; and SD - Standard deviation.				

Supplementary Table 2. Functional annotation of modules associated with HIV

Modules	Size	Biological pathways	P-value (Bonf adj)	Databases
Modules positively associated with HIV				
PM37	428	Response to odorant<br>Branching morphogenesis of a nerve<br>Regulation of type 2 immune response	ns<br>ns<br>0.04	DAVID<br>WebGestalt<br>STRING
PM31	185	Negative regulation of natural killer cell chemotaxis<br>Exploration behaviour<br>Signaling receptor regulator activity	ns<br>ns<br>ns	DAVID<br>WebGestalt<br>STRING
PM5	22	Type B pancreatic cell development<br>Glandular epithelial cell development	ns<br>ns	DAVID<br>WebGestalt
PM16	72	Cellular potassium ion homeostasis<br>Negative regulation of necroptotic process	ns<br>ns	DAVID<br>WebGestalt
PM6	23	Very-low density lipoprotein particle assembly<br>Lipoprotein particle binding	ns<br>ns	WebGestalt<br>STRING
PM8	30	Complement activation<br>Complement activation<br>Cytolysis	**0.01**<br>**0.001**<br>**0.02**	DAVID<br>WebGestalt<br>STRING
PM39	1203	Translational initiation<br>Establishment of localization in cell<br>Peptide transport*<br>Regulation of Golgi inheritance	**0.0000**<br>**0.0001**<br>**0.0001**	DAVID<br>WebGestalt<br>DAVID<br>STRING
PM2	12			
Modules negatively associated with HIV				
PM17	73	Cysteine biosynthetic process from serine<br>Regulation of high voltage-gated calcium channel activity	ns<br>ns	DAVID<br>WebGestalt
PM38	665	Synaptic membrane adhesion<br>Axonogenesis<br>Positive regulation of cardiac neural crest migration invasion	**0.000**<br>**0.000**<br>**0.01**	DAVID<br>WebGestalt<br>STRING
PM14	66	Regulation of heparan sulfate proteoglycan biosynthetic process<br>Glial cell fate commitment	ns<br>ns	DAVID<br>WebGestalt
PM32	218	Purine nucleotide biosynthesis process<br>5-phosphoribose 1-diphosphate biosynthetic process	**0.000**<br>**0.000**	DAVID/STRING<br>WebGestalt
PM30	176	Innate immune response<br>Positive regulation of immune effector process	**0.04**<br>**0.0003**	WebGestalt<br>STRING
PM10	30	Sulfation	ns	WebGestalt
PM25	124	Carbohydrate phosphorylation	ns	WebGestalt
PM33	224	Ribosomal small subunit biogenesis<br>mRNA processing<br>Cellular response to sodium arsenite	**0.000**<br>**0.000**<br>**0.009**	DAVID<br>WebGestalt<br>STRING
PM34	266	Notch receptor processing<br>Défense response to other organism	ns<br>0.02	WebGestalt<br>STRING
PM1	11	Regulation of blood coagulation<br>Blood coagulation<br>Negative regulation of fibrinolysis	**0.004**<br>**0.000**<br>**0.013**	DAVID<br>WebGestalt<br>STRING
PM12	48	Immature T cell proliferation in thymus<br>Mitotic cell cycle phase transition	ns<br>**0.040**	DAVID<br>WebGestalt
PM18	74	Regulation of activin receptor signalling pathway<br>Synapse organization<br>Proximal/distal axis specification<br>Regulation of activin receptor signalling pathway	ns<br>ns<br>**0.04**<br>**0.04**	DAVID<br>WebGestalt<br>STRING<br>STRING
PM27	155	Positive regulation of mitotic sister chromatid separation<br>Chondrocyte proliferation<br>Positive regulation of peptidyl-tyrosine phosphorylation	ns<br>ns<br>**0.03**	DAVID<br>WebGestalt<br>STRING
PM3	15	Negative regulation of proteolysis<br>Blood coagulation, intrinsic pathway	ns<br>ns	DAVID<br>WebGestalt
PM7	25	Insulin-like growth factor receptor signalling pathway<br>Positive regulation of insulin-like growth factor receptor signalling pathway	**0.02**<br>**0.01**	DAVID/STRING<br>WebGestalt

PM19	79	Negative regulation of DNA damage checkpoint<br>Hindlimb morphogenesis<br>Chondroblast differentiation and Hindlimb morphogenesis	ns<br>**0.02**<br>**0.03**	DAVID<br>WebGestalt<br>STRINNG
PM26	125	Collagen fibril organization<br>Bone morphogenesis<br>Bone trabecular formation	**0.000**<br>**0.000**<br>**0.007**	DAVID<br>WebGestalt<br>STRING
PM22	83	Telomere organization<br>Chromatin assembly<br>Leukocyte mediated cytotoxicity	**0.001**<br>**0.007**<br>**0.02**	DAVID<br>WebGestalt<br>STRING
PM23	84	Uterine wall breakdown<br>Regulation of signalling receptor activity<br>Positive regulation of T-helper 2 cell cytokine production	ns<br>**0.001**<br>**0.01**	DAVID<br>WebGestalt<br>STRING
Superclusters				
SC1-PM17,30,34	513	Inflammatory response	**0.03**	DAVID
SC2-PM19,22,23	319	Positive regulation of T-helper 2 cell cytokine production<br>Regulation of signalling receptor activity	**0.0001**<br>**0.0001**	DAVID<br>WebGestalt
SC3-PM12,32,33	489	Ribosomal small subunit biosynthesis<br>mRNA metabolic process	**0.0001**<br>**0.0001**	DAVID<br>WebGestalt
SC4-PM25,10	154	Response to interleukin-4	ns	DAVID
SC5-PM7,8	55	Complement activation<br>Regulation of humoral immune response	**0.0001**<br>**0.0001**	DAVID<br>WebGestalt
SC6-PM31,37	613	Humoral immune response*	**0.02**	DAVID

*Homo sapiens served as the reference background for calculating fold enrichment for Gene Ontology (GO) enrichment analysis for biological processes using DAVID, STRING, and WebGestalt databases. Enrichment was assessed with Fisher's exact or hypergeometric tests, and Bonferroni-adjusted p-values were used to determine significance. Abbreviation: ns = not significantly enriched biological pathway.

Supplementary Table 3. Candidate hub proteins within modules significantly associated with HIV status

Modules	Hub protein	Description	UniProt/STRING functional annotation	HIV literature on these hub proteins from PubMed
**Modules positively associated with HIV**				
PM5	TRAPPC3	Trafficking protein particle complex subunit 3 (TRAPPC3)	As a component of TRAPP complex, TRAPPC3 could be involved in vesicular transport from endoplasmic reticulum to Golgi.<br><br>Vesicle tethering§	TRAPPC1 a component of TRAPP complex is required for HIV infection[1]. Lower expression of HIV-permissive host genes including TRAPPC1 were associated with deceased susceptibility to HIV-1 infection in female sex workers[2].
PM6	SC5A8	Sodium-coupled monocarboxylate transporter 1	Acts as a sodium ion-coupled solute transporter of short-chain fatty acids, monocarboxylates drugs and ketone bodies.<br><br>Positive regulation of acute inflammatory response to non-antigenic stimulus§	Transport of ions including lactate across the cell membrane is aided by monocarboxylates transporters (MCTs) whose direction is determined by the concentration of monocarboxylates ions and protons. Similarly, sodium coupled transport is also performed by SC5A8. The levels of concentration of these transporters is affected by inflammation. Solute carrier transporters such GLUT1 have been implicated in HIV infection. GLUT1, a primary glucose transporter in lymphocytes is increased in CD4+ and CD8+ T cells in HIV infection[3].
PM8	CFHR5	Complement factor H-related protein 5	Involved in complement regulation.<br><br>Opsonization§	In adults living with HIV on ART, CFHR5 was significantly enriched compared to their seronegative counterparts. CFHR5 exhibited borderline association with prevalence of non-AIDS related comorbidities[4].
PM16	FGF-8B	Fibroblast growth factor 8 isoform B	FGF8b is one of the four isoforms of FGF. It is involved in embryonic development, cell proliferation and migration. Required for normal development of the gonadotropin-releasing hormone neural system.<br><br>Prostate epithelial cord elongation§	
PM31	SEM4F	Semaphorin-4F	A probable cell surface receptor that regulates oligodendroglial precursor cell migration. Can also regulate differentiation of oligodendroglial precursor cells.<br><br>Olfactory nerve formation§	
PM37	DNS2B	Deoxyribonuclease-2-beta	Preferentially hydrolyses double stranded DNA under acidic conditions. Plays a role in the clearance of nucleic acids generated through apoptosis, hence preventing autoinflammation.	

			DNA catabolic process, endonucleolytic§	
PM39	SH3G2	Endophilin-A1	Implicated in the synaptic vesicle endocytosis. Required for BDNF-dependent dendrite outgrowth.<br><br>Synaptic vesicle uncoating§	SH3 domain mediates the binding of Tat protein with human proteins including Grb2. The binding of Tat to Grb2 impairs activation of MAPK/Raf pathway. This interaction also affects viral function by inhibiting the Tat-mediated transactivation of HIV-1 long terminal repeats and viral replication in infected primary microglia[5].
**Modules negatively associated with HIV**				
PM1	Transferrin	Serotransferrin	Binds and transport iron in the body. Elevated transferrin used as a marker of iron deficiency<br><br>Cellular response to iron ion§	HIV has been associated with low transferrin levels[6].
PM3	KNG2	Kininogen, HMW, Two Chain	Play a role in coagulation.<br><br>Positive regulation of fibrinolysis§	HIV^+ patients with and without pruritic papular eruption (skin rashes) had higher plasma levels of total kininogen (high molecular weight and low molecular weight kininogen) than their HIV^- controls. HMWK levels were diminished in HIV^+ group although without significance when compared to their counterparts[7].
PM7	IGFBP-3	Insulin-like growth factor-binding protein 3	Binds and regulate the actions of IGF-I. IGFBP-3 is the main transporter of IGFs in the bloodstream.<br><br>Positive regulation of insulin-like growth factor receptor signalling pathway§	Infection with HIV is often associated with a decrease in the concentrations of IGF-I, IGF-II, IGFBP-3 and increase of IGFBP-1 and -2. IGFBP-3 protease activity in the serum is increased during conditions such as protein deprivation and chronic illness e.g., HIV infection[8,9].
PM10	CCD43	Coiled-coil domain-containing protein 43	Protein involved in acetylation§	
PM12	MOB3B	MOB kinase activator 3B	Regulates large tumor suppressor kinase 1 (LATS1) expression in the Hippo pathway which plays a crucial role in organ size control and tumor suppression by promoting apoptosis and inhibiting proliferation.<br><br>Inner cell mass cellular morphogenesis§	
PM14	DPH5	Diphthine methyl ester synthase	Involved in post translational methylation. DPH5 gene is involved in diphthamide biosynthesis.<br><br>Protein histidyl modification to diphthamide§	Diphthamide is a cellular target in viral infection. It ensures maintenance of reading frame and its deficiency is linked to increased -1 programmed ribosomal frameshifting propagating viral infection. DPH7 one of the diphthamide synthase enzyme has been associated with targeted vif protein degradation thus promoting frameshifting necessary for HIV infection[10].
PM17	MBL2	Vesicular integral-membrane protein VIP36	Plays a role as an intracellular lectin in the early secretory pathway. Involved in transport and	

			sorting of glycoproteins carrying high mannose-type glycans.<br><br>COPI coating of Golgi vesicle§	
PM18	CA7	Carbonic anhydrase 7	Involved in catalytic reversible hydration of carbon dioxide.<br><br>Positive regulation of cellular pH reduction§	
PM19	SOD	Superoxide dismutase	An antioxidant enzyme that protects the cell from reactive oxygen species (ROS) toxicity.<br><br>Positive regulation of oxidative stress-induced intrinsic apoptotic signalling pathway§	SOD controls ROS and oxidative stress[11]. HIV infection and the Tat protein can induce ROS production and alter redox regulation[12]. In prepubertal children with and without HIV similar SOD levels were observed [13]. In adults living with HIV, SOD levels were elevated in physically active group compared to the inactive one[14].
PM22	H2B2E	Histone H2B type 2-E	Is a core component of nucleosome. Nucleosomes wrap and compact DNA into chromatin, limiting its accessibility to the cellular machineries which depend on DNA as a template.<br><br>Protein localization to chromosome, centromeric region§	
PM23	Enterokinase	Enteropeptidase	Responsible for initiating activation of pancreatic proteolytic proenzymes. It catalyses the conversion of trypsinogen to trypsin which in turn activates other proenzymes such as proelastases, procarboxypeptidases and chymotrypsinogen.	An in vitro study investigating the effects of HAART drugs and excipient on the activity of digestive enzymes reported no association between HAART drug formulations and inhibition of trypsin or enterokinase activity[15].
PM25	CQ10A	Coenzyme Q-binding protein COQIO homolog A, mitochondrial	Required for coenzyme Q functioning in the mitochondrial respiratory chain.<br><br>Ubiquinone biosynthesis process§	A clinical trial assessing the effect of COQ10 supplementation on incidences of opportunistic infections and levels of CD4^{+} T-cell counts among HIV-infected adults reported no improvement in these measurements[16].
PM26	COL11A2	Collagen alpha-2(XI) chain	Involved in fibrillogenesis by controlling lateral growth of collagen II fibrils. Type XI collagen is mainly found in cartilage.<br><br>Negative regulation of endodermal cell differentiation§	
PM27	FR1OP	FGFR1 oncogene partner	Is a centrosomal protein involved in anchoring microtubules to the centromeres.<br><br>Microtubule anchoring§	
PM30	OPT	Opticin	Inhibits angiogenesis in the vitreous humour of	

			the eye thereby repressing neovascularization. May be involved in collagen fibre organization through regulation of other members of the small leucine-rich repeat proteoglycan superfamily<br><br>Collagen catabolic process§	
PM32	FGF-16	Fibroblasts growth factor 16	Plays a role in the regulation of embryo development, cell proliferation. Required for normal cardiomyocyte proliferation and heart development.<br><br>Positive regulation of endothelial cell chemotaxis to fibroblast growth factor§	In a cell line study model that investigated the interactions of neurotrophins and HIV induced macrophage activation, reported altered growth factor secretion where FGF16 levels were decreased in cells exposed to HIV[17].
PM33	STMN4	Stathmin-4	Exhibits microtubule-destabilizing activity. Prevents assembly and promotes disassembly of microtubules.<br><br>COPII vesicle coating§	Stathmin-1 a protein also involved in the regulation of the microtubule filament by destabilizing microtubules has been associated with the maintenance of HIV-1 latency[18].
PM34	SATB1	DNA binding protein SATB1	Transcriptional repressor controlling nuclear and viral expression in a phosphorylated and acetylated dependent manner.<br><br>Facultative heterochromatin formation§	STAB1 was found to be positively associated with markers of immune activation among HIV$^+$ individuals on ART in South Africa[19].
PM38	EPHA4	Ephrin type-A receptor 4	Involved in development of nervous system.<br><br>Positive regulation of aspartic-type endopeptidase activity involved in amyloid precursor protein catabolic process§	Lower levels of EPHA4 transcripts were associated with HIV-related neurocognitive disorders among HIV$^+$ adults on ART compared to the HIV$^-$ group[20].

§ Biological process functional annotation (Gene Ontology) from STRING database

Supplementary Table 4. Top hub proteins with high effect sizes and intra-modular connectivity within modules associated with HIV

Modules	Target	Description	Within-module Connectivity	Effect size
PM1	a1-Antichymotrypsin	Alpha-1-antichymotrypsin	1	0.085
PM1	Transferrin	Serotransferrin	0.885	0.126
PM1	Prothrombin	Prothrombin	0.723	0.069
PM1	Kininogen, HMW	Kininogen-1	0.649	0.083
PM3	TPGS2	Tubulin polyglutamylase complex subunit 2	1	0.152
PM3	HSP 70	Heat shock 70 protein 1A	0.994	0.141
PM3	KNG2	Kininogen, HMW, Two Chain	0.892	0.16
PM3	CGAT2	Chondroitin sulfate N acetylgalactosaminyltransferase 2	0.6	0.135
PM3	IL-1b	Interleukin-1 beta	0.558	0.123
PM5	ERP29	Endoplasmic reticulum resident protein 29	1	0.056
PM5	HNF4A	Hepatocyte nuclear factor 4-alpha	0.871	0.082
PM5	a1-Microglobulin	Alpha-1-microglobulin	0.813	0.083
PM5	TPPC3	Trafficking protein particle complex subunit 3	0.786	0.115
PM5	SUMF1	Sulfatase-modifying factor 1	0.673	0.156
PM5	DLL1	Delta-like protein 1	0.588	0.064
PM7	IGF-I	Insulin-like growth factor I	1	0.17
PM7	IGFBP-3	Insulin-like growth factor-binding protein 3	0.956	0.175
PM7	IGFALS	IGF binding protein complex acid labile subunit	0.756	0.173
PM8	CFHR5	Complement factor H related 5	0.973	0.101
PM8	CAD15:CD	Cadherin-15:Cytoplasmic domain	0.899	0.117
PM8	PXDC1	Plexin domain-containing protein 1	0.715	0.08
PM8	VCX1	Variable charge X-linked protein 1	0.632	0.067
PM8	GON1	Progonadoliberin-1	0.622	0.104
PM12	MOB3B	MOB kinase activator 3B	0.998	0.203
PM12	ITM2A	Integral membrane protein 2A	0.99	0.107
PM12	HDAC8	Histone deacetylase 8	0.977	0.098
PM12	PDCL2	Phosducin-like protein 2	0.848	0.151
PM12	RGS1	Regulator of G-protein signaling 1	0.755	0.166
PM12	EGLN2	Egl nine homolog 2	0.716	0.081
PM12	BR serine/threonine kinase 2	Serine/threonine-protein kinase BRSK2	0.698	0.09
PM12	Megalin	Low-density lipoprotein receptor-related protein 2	0.668	0.09
PM12	CUL1	Cullin-1	0.654	0.105
PM14	DPH5	Diphthine methyl ester synthase	1	0.095
PM14	IFT22	Intraflagellar transport protein 22 homolog	0.729	0.052
PM14	FUT9	Alpha-(1,3)-fucosyltransferase 9	0.655	0.06
PM14	ZCC18	Zinc finger CCHC domain-containing protein 18	0.618	0.115
PM16	FGF8B	Fibroblast growth factor 8 isoform B	1	0.118
PM16	OLR1	Oxidized low-density lipoprotein receptor 1	0.928	0.1
PM16	Collagen alpha-3(VI):isoform 3	Collagen alpha-3(VI) chain:isoform 3	0.922	0.108
PM16	NNAT	Neuronatin	0.919	0.109
PM16	ARL14	ADP-ribosylation factor-like protein 14	0.862	0.119
PM16	CPTP	Ceramide-1-phosphate transfer protein	0.857	0.104
PM16	Semaphorin 3E	Semaphorin 3E	0.817	0.126

PM16	Beta-casein	Beta-casein	0.723	0.088
PM16	5NT1A	Cytosolic 5'-nucleotidase 1A	0.723	0.129
PM16	MAGE-4	Melanoma-associated antigen 4	0.718	0.101
PM16	MAP3K3	Mitogen-activated protein kinase kinase kinase 3	0.71	0.097
PM17	MBL2	Mannose binding lectin 2	1	0
PM17	CHKB	Choline/ethanolamine kinase	0.987	0.017
PM17	OLFL3	Olfactomedin-like protein 3	0.933	0.026
PM17	PCDGK	Protocadherin gamma-C3	0.777	0.002
PM17	SIG12:Ig-like C2-type 2	Sialic acid-binding Ig-like lectin 12:Ig-like C2-type 2 domain, Isoform short	0.719	0.025
PM18	LRRT3	Leucine rich repeat transmembrane neuronal protein 3	0.934	0.108
PM18	CA7	Carbonic anhydrase 7	0.896	0.156
PM18	IL-20 Ra	Interleukin-20 receptor subunit alpha	0.85	0.068
PM18	MRP6	Multidrug resistance-associated protein 8	0.85	0.068
PM18	GPC2	Glypican-2	0.742	0.066
PM19	SOD	Superoxide dismutase [Cu-Zn]	1	0.12
PM19	Azurocidin	Azurocidin	0.777	0.102
PM19	MFRP	Membrane frizzled-related protein	0.768	0.089
PM19	Activin AB	Inhibin beta A chain:Inhibin beta B chain heterodi mer	0.753	0.108
PM19	Bone proteoglycan II	Decorin	0.727	0.156
PM19	TBK1	Serine/threonine-protein kinase	0.712	0.167
PM19	TSLP	Thymic stromal lymphopoietin	0.706	0.12
PM19	RSPO2	R-spondin-2	0.689	0.204
PM19	IL-11	Interleukin-11	0.637	0.151
PM19	MED-1	Mediator of RNA polymerase II transcription subu nit 1	0.629	0.098
PM19	CD47	Leukocyte surface antigen CD47	0.628	0.128
PM19	FST	Follistatin	0.62	0.092
PM22	GFRa-3	GDNF family receptor alpha-3	1	0.144
PM22	CELF2	CUGBP Elav-like family member 2	0.997	0.128
PM22	H2B1K	Histone H2B type 1-K	0.993	0.152
PM22	H2B3B	Histone H2B type 3-B	0.955	0.122
PM22	H2B2E	Histone H2B type 2-E	0.913	0.168
PM22	H2A3	Histone H2A type 3	0.904	0.154
PM22	IL22RA1	Interleukin-22 receptor subunit alpha-1	0.899	0.145
PM22	H2A1A	Histone H2A type 1-A	0.832	0.118
PM22	H2B2E	Histone H2B type 2-E	0.826	0.129
PM22	Histone H2A type 1	Histone H2A type 1	0.759	0.162
PM23	IFNL2	Interferon lambda-2	1	0.11
PM23	Enterokinase	Enteropeptidase	0.955	0.114
PM23	CHST6	Carbohydrate sulfotransferase 6	0.917	0.114
PM23	WFKN1	WAP, kazal, immunoglobulin, kunitz and NTR do main-containing protein 1	0.917	0.114
PM23	PESC	Pescadillo homolog	0.84	0.111
PM23	ABL1	Tyrosine-protein kinase ABL1	0.824	0.083
PM23	PLK-1	Serine/threonine-protein kinase PLK1	0.745	0.079
PM23	NANOG	Homeobox protein NANOG	0.735	0.089
PM23	ANP	Atrial natriuretic factor	0.723	0.116
PM23	Lymphotactin	Lymphotactin	0.72	0.094
PM26	COL11A2	Collagen alpha-2(XI) chain	1	0.14
PM26	CO9A1	Collagen alpha-1(IX) chain	0.979	0.113
PM26	TSP4	Thrombospondin-4	0.927	0.117

PM26	TSP3	Thrombospondin-3	0.857	0.115
PM26	CILP2	Cartilage intermediate layer protein 2	0.841	0.125
PM26	LRC15	Leucine-rich repeat-containing protein 15	0.804	0.116
PM26	CO1A1:C-term propeptide	Collagen alpha-1(I) chain:C-term propeptide	0.781	0.101
PM26	HPLN1	Hyaluronan and proteoglycan link protein 1	0.733	0.086
PM26	CNTN3	Contactin-3	0.715	0.108
PM26	DERM	Dermatopontin	0.705	0.121
PM27	MSP R	Macrophage-stimulating protein receptor	1	0.079
PM27	ENTP3	Ectonucleoside triphosphate diphosphohydrolase 3	0.998	0.061
PM27	TMM46	Protein shisa-2 homolog	0.938	0.073
PM27	TMCC3:region 2	Transmembrane and coiled-coil domains protein 3: region 2	0.935	0.065
PM27	PECAM-1	Platelet endothelial cell adhesion molecule	0.86	0.132
PM27	AURKB	Aurora kinase B	0.857	0.102
PM27	CAMK1D	Calcium/calmodulin-dependent protein kinase type 1D	0.852	0.075
PM27	CD22	B-cell receptor CD22	0.82	0.12
PM27	TMA	Thyroid peroxidase	0.804	0.076
PM27	KI16B	Kinesin-like protein KIF16B	0.788	0.113
PM27	CCNB2	G2/mitotic-specific cyclin-B2	0.786	0.066
PM27	FR1OP	FGFR1 oncogene partner	0.78	0.157
PM31	SEM4F	Semaphorin-4F	0.798	0.001
PM31	XLRS1	Retinoschisin	0.779	0.008
PM31	cAMP-regulated phosphoprotein 21	cAMP-regulated phosphoprotein 21	0.778	0.024
PM31	KLRF1	Killer cell lectin-like receptor subfamily F member 1	0.727	0.011
PM31	IFNA6	Interferon alpha-6	0.715	0.001
PM31	TM11D	Transmembrane protease serine 11D	0.683	0.01
PM31	SESQ2	Sesquipedalian-2	0.682	0.005
PM31	FA24B	Protein FAM24B	0.676	0.004
PM31	YMEL1	ATP-dependent zinc metalloprotease YME1L1	0.668	0.006
PM31	UROL1	Uromodulin-like 1	0.662	0.003
PM36	GNT2C	N-acetyllactosaminide beta-1,6-N-acetylglucosaminyl-transferase, isoform C	1	0.003
PM36	KELL	Kell blood group glycoprotein	0.987	0.004
PM36	LIMP II	Lysosome membrane protein 2	0.947	0.03
PM36	KLHL3	Kelch-like protein 3	0.938	0.031
PM36	DPP10	Inactive dipeptidyl peptidase 10	0.912	0.022
PM36	SIM13	Small integral membrane protein 13	0.888	0.009
PM36	PRDX4	Peroxiredoxin-4	0.861	0.002
PM36	TUTLB	Protein turtle homolog B	0.838	0.035
PM36	OBP2A	Odorant-binding protein 2a	0.824	0.004
PM36	FABP6	Gastrotropin	0.803	0.002
PM36	GALT3	Polypeptide N-acetylgalactosaminyltransferase 3	0.801	0.032
PM37	SPEF1	Sperm flagellar protein 1	1	0.108
PM37	NGRN	Neugrin	0.995	0.102
PM37	SORC3	VPS10 domain-containing receptor SorCS3	0.979	0.094
PM37	DNS2B	Deoxyribonuclease-2-beta	0.957	0.134
PM37	ISL1	Insulin gene enhancer protein ISL-1	0.948	0.113
PM37	VAPB	Vesicle-associated membrane protein- associated protein B/C	0.944	0.13
PM37	HCCR-1	LETM1 domain-containing protein 1	0.916	0.108
PM37	COPA1	Collagen alpha-1(XXV) chain	0.906	0.114

PM37	PLD5	Inactive phospholipase D5	0.905	0.117
PM37	SNX1	Sorting nexin-1	0.896	0.1
PM37	IDD	Integral membrane protein DGCR2/IDD	0.893	0.093
PM37	MOT4	Monocarboxylate transporter 4	0.891	0.134
PM38	EPHA4	Ephrin type-A receptor 4	1	0.127
PM38	ISLR2	Immunoglobulin superfamily containing leucine-rich repeat protein 2	0.863	0.095
PM38	ROBO2	Roundabout homolog 2	0.836	0.106
PM38	UNC5B	Netrin receptor UNC5B	0.826	0.098
PM38	ISLR2	Immunoglobulin superfamily containing leucine-rich repeat protein 2	0.803	0.084
PM38	ROR1	Inactive tyrosine-protein kinase transmembrane receptor ROR1	0.801	0.117
PM38	T132D	Transmembrane protein 132D	0.797	0.097
PM38	LRRT2	Leucine<br>rich repeat transmembrane neuronal protein 2	0.792	0.08
PM38	FLRT2	Leucine-rich repeat transmembrane protein FLRT2	0.785	0.126
PM38	EPHA4	Ephrin type-A receptor 4	0.775	0.122

Supplementary Table 5. Modules and candidate hub proteins within modules significantly associated with 90-day post discharge growth

**MUAC**				
**Modules**	**Target**	**Protein**	**UniProt functional annotation**	**Growth literature on these hub proteins - PubMed**
PM12	ITM2A	Integral membrane protein 2A	In mouse models, postulated to be involved in osteo- and chondrogenic differentiation.<br><br>Hematopoietic cell lineage[§]	In mouse model, ITM2A was found to be expressed in differentiated muscles, hair the follicles in skin and in diaphragm. In these tissues, ITM2A expression also increased during embryonic development. Additionally, ITM2A was expressed at the onset of chondrocytic differentiation in growth plates[21]. An in vitro study demonstrated that overexpression of IMT2A enhanced myogenic differentiation[22].
PM26	CO9A1	Collagen alpha-1(IX) chain	Structural component of hyaline and vitreous of the eye.<br><br>Extracellular matrix organization[§]	Collagen IX is a stabilizing component of the cartilage fibrils. A mouse model study demonstrated that absence of collagen IX seriously compromised growth cartilage structure and differentiation in mice. Specifically, changes in growth plate morphology were prominent in late proliferative, pre-hypertrophic and hypertrophic zones. Also, in the epiphyseal regions of long bones, new-borns to the mice lacking collagen IX had reduced cell numbers, irregular distribution of glycosaminoglycan in the ECM and disturbed columnar arrangement of chondrocytes which made to long bones to be shorter and broader in new-borns[23].
PM31	ARPP21	cAMP-regulated phosphoprotein 21	Involved in the integration of key neurotransmitter inputs into medium spiny neurons through its regulation of calmodulin-dependent kinase-1 and protein phosphatae-2B.<br><br>Negative regulation of protein dephosphorylation[§]	
PM37	NGRN	Neugrin	Plays vital role in mitochondrial ribosome biogenesis.<br><br>Mitochondrial ribosome assembly[§]	
**WAZ**				
PM7	IGF-I	Insulin-like growth factor 1	Involved in growth and development.<br><br>Regulation of insulin-like growth factor receptor signalling pathway[§]	IGF-1 has been mainly associated with postnatal growth. In mutant animal models where IGF-1 action was impaired, most animals died shortly after birth and those that survived had growth defects[24,25]. In human, poor nutritional status is associated with reduced levels of IGF-1 in blood among undernourished children[26,27].
PM26	CILP2	Cartilage intermediate layer protein 2	May play a role in cartilage scaffolding.	In adults, serum levels of CLIP2 in overweight and obese individuals

			Glycosaminoglycan binding§	was higher than that in individuals with normal BMI values[28].
PM31	SMDC1	SAYSvFN domain-containing protein 1		
PM37	ISL1	Insulin gene enhancer protein ISL-1	DNA binding transcriptional activator. Regulates the expression of insulin, glucagon, somatostatin, and pancreatic polypeptides in postanal islet tissue.<br><br>Medial motor column neuron differentiation§	In murine study models, reduced expression of ISL-1 inhibited proliferation, migration and tube formation in vascular endothelial cells[29].
**WHZ**				
PM7	IGF-I	Insulin-like growth factor 1	Involved in growth and development.<br><br>Regulation of insulin-like growth factor receptor signalling pathway§	IGF-1 levels are responsive to both acute and chronic malnutrition[26,27].
PM26	TSP3	Thrombospondin-3	In a mouse model it is an adhesive protein that mediates cell-to-cell and cell-to-matrix interactions. Can bind fibrinogen, fibronectin, laminin and type V collagen.<br><br>Positive regulation of extracellular exosome assembly§	TSP3 expression has been detected in the developing skeleton and in adult bone, particularly in the early proliferative zone of the growth plate of long bones, where it is expressed by chondrocytes[30]. TSP3 knockout mice exhibited growth plate abnormalities[31].
PM31	BRD2	Bromodomain-containing protein 2	Involved in chromatin remodelling and nucleosome assembly. Binds hyperacetylated chromatin thus regulating transcription of CCND1 gene.<br><br>Positive regulation of histone H3-K36 trimethylation§	In cell line model study, bromodomain and extra-terminal protein (BET) inhibitors impeded chondrogenesis, a biological process through which bones are formed. BRD2 is one of the protein members that constitute BET. Mechanistically BET inhibitors influenced the depletion of RNA polymerase II from the *col2al* promoter. Type II collagen, *col2al* is important for chondrocyte maintenance and proliferation[32].
PM33	STMN4	Stathmin-4	Exhibits microtubule-destabilizing activity.<br><br>COPII vesicle coating§	
PM37	ISL1	Insulin gene enhancer protein ISL-1	DNA binding transcriptional activator. Regulates the expression of insulin, glucagon, somatostatin, and pancreatic polypeptides in postanal islet tissue.<br><br>Medial motor column neuron differentiation§	In murine study models, reduced expression of ISL-1 inhibited proliferation, migration and tube formation in vascular endothelial cells[29].
**HAZ**				
PM3	TPGS2	Tubulin polyglutamylase complex subunit 2	Involved in post-translational addition of glutamate residues to C-terminal tubulin tails.<br><br>Protein polyglutamylation§	
PM12	MOB3B	MOB kinase activator 3B	Regulates large tumor suppressor kinase 1 (LATS1) expression in the Hippo pathway which plays a crucial role in organ size	

			control and tumor suppression by promoting apoptosis and inhibiting proliferation.<br><br>Inner cell mass cellular morphogenesis§	
PM18	LRRT3	Leucine-rich repeat transmembrane neuronal protein 3	May be involved in development and maintenance of nervous system.<br><br>Neuroligin clustering involved in postsynaptic membrane assembly§<br><br>Regulation of activin receptor signalling pathway§	

§ Biological process functional annotation (Gene Ontology) from STRING database

Supplementary Methods

Equations for computing weights using inverse probability weighting (IPW)

$$\text{HIV status} = \text{age} + \text{sex} + \text{malaria} + \text{diarrhoea} + \text{pneumonia} + \text{nutritional status} + (1|\text{site}) + u \quad (1)$$

$$\text{Weights} = \text{predict(HIV status, type} = \text{“response”)} \quad (2)$$

$$\text{IPW} = 1/Weights \text{ (for children with HIV)}; = 1/(1 - Weights) \text{ (for children without HIV)} \quad (3)$$

This was implemented using the glmer function in lme4 package in R. The codes used for generating the weights using IPW are available at Harvard Dataverse website using this link https://doi.org/10.7910/DVN/D8HZLJ or can be found at https://github.com/mudiboevans/HIV-SM-PROTEOMICS[33].

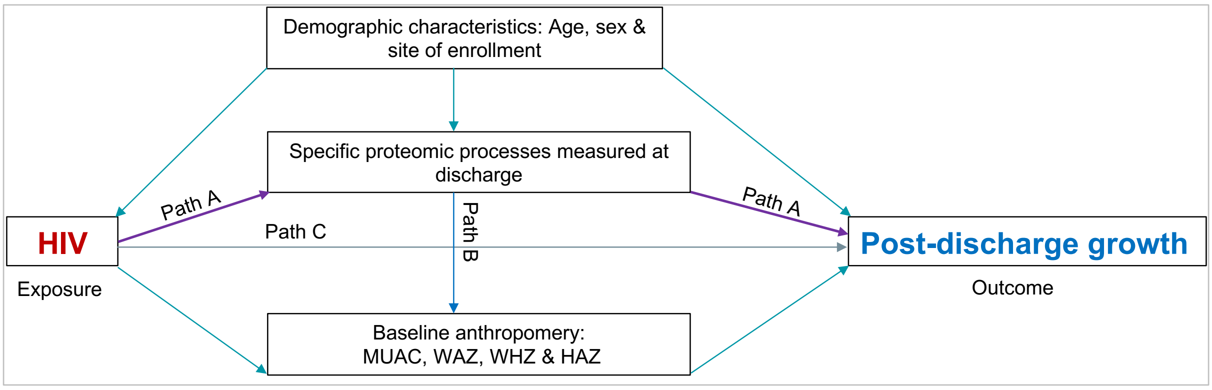

Supplementary Figure 5. Hypothesised path model linking HIV to post-discharge growth. The proposed pathway framework outlines link between HIV and post-discharge growth in children. The model incorporates site as a potential influencing factor, recognizing variations in HIV prevalence among different locations. Age, which may impact the child's viral load, is also integrated into the model. The hypothesised path model suggests that HIV indirectly affects post-discharge growth through modulation of certain biological processes measured at hospital discharge in human blood plasma, as denoted by the purple-coloured arrow (path A). This framework serves as a guide for analysing the intricate biological relationship between HIV and post-discharge growth in children.

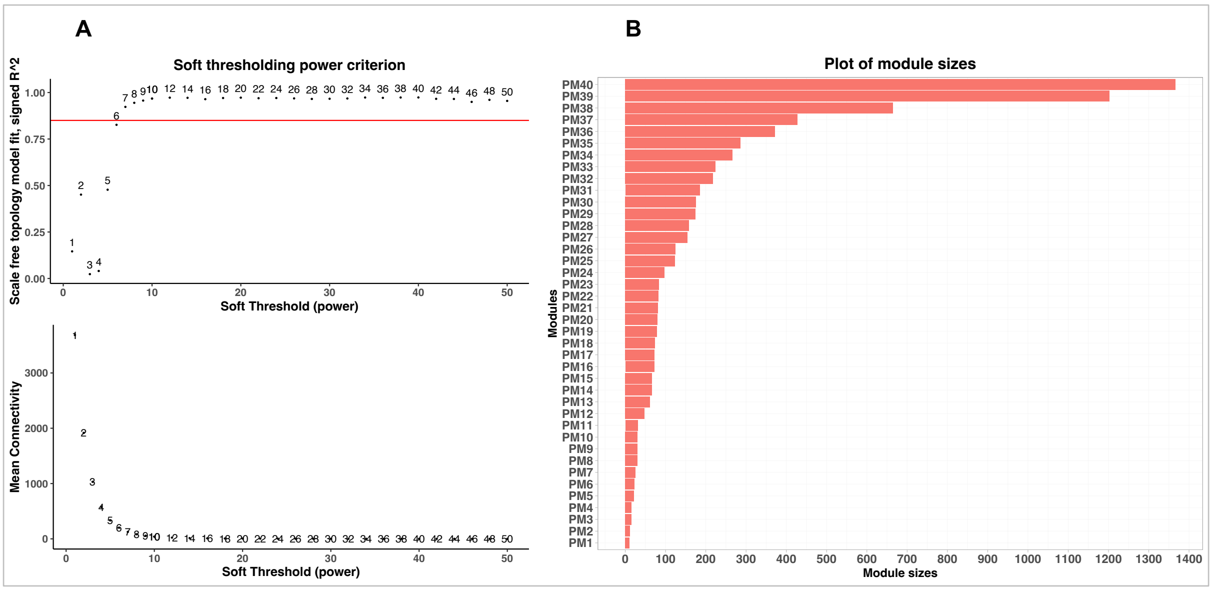

Supplementary Figure 6. Selection of an optimal power for generation of the network. (A). Scale-free topology criterion. A soft thresholding power of 9 was chosen to genearate a network of proteins that assumes a scale-free topography. The power of 9 was selected based on r-squared of 0.85 (red line in the top panel) and minimum mean connectivity (bottom panel). **(B)**. Bar plot showing module sizes (number of proteins in every module) from a soft thresholding power of 9 , the smallest module has 11 proteins (PM1) while the largest module contains 1203 proteins (PM39). PM40 contains 1366 unassigned proteins. This module was not considered in the subsequent analysis as it contains unsassigned proteins.

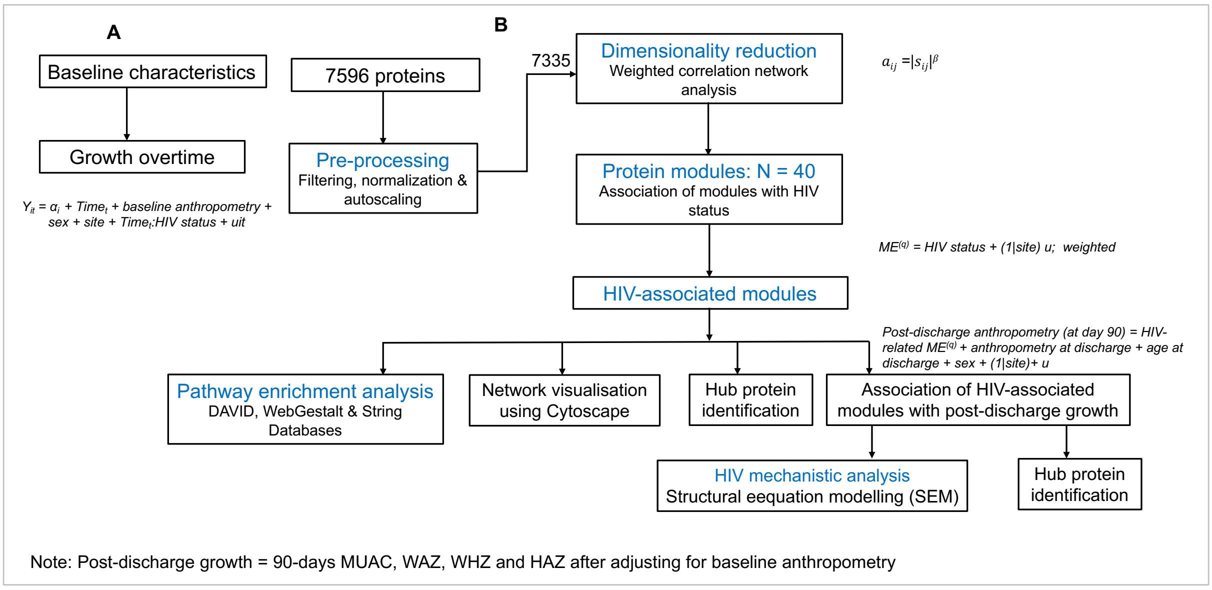

Supplementary Figure 7. A general analysis workflow of the present study. (A). Analysis of participants characteristics and growth from discharge through six months post-discharge. The figure demonstrates the analyses steps including the profiling of baseline characteristics of the partcipants, evaluation of the growth overtime by HIV status. (B). Involves proteomic data analysis; pre-processing, dimensionality reduction through weighted correlation network analysis (construction of the plasma proteome network), identification of individual protein modules and their association with HIV status,

association of HIV-related protein modules with 90-day post-discharge growth, identification of hub proteins within individual modules, network visualization and enrichment analysis to identify over-represented biological process. Lastly, identification of potential directed paths through which HIV influences post-discharge growth using structural equation modelling (SEM).

Supplementary References

1 Brass, A. L. *et al.* Identification of host proteins required for HIV infection through a functional genomic screen. *Science* **319**, 921-926 (2008). https://doi.org:10.1126/science.1152725

2 Abdulhaqq, S. A. *et al.* HIV-1-negative female sex workers sustain high cervical IFNε, low immune activation, and low expression of HIV-1-required host genes. *Mucosal Immunol* **9**, 1027-1038 (2016). https://doi.org:10.1038/mi.2015.116

3 Song, W., Li, D., Tao, L., Luo, Q. & Chen, L. Solute carrier transporters: the metabolic gatekeepers of immune cells. *Acta Pharm Sin B* **10**, 61-78 (2020). https://doi.org:10.1016/j.apsb.2019.12.006

4 Vujkovic-Cvijin, I. *et al.* The Complement Pathway Is Activated in People With Human Immunodeficiency Virus and Is Associated With Non-AIDS Comorbidities. *J Infect Dis* **224**, 1405-1409 (2021). https://doi.org:10.1093/infdis/jiab096

5 Rom, S. *et al.* HIV-1 Tat binds to SH3 domains: cellular and viral outcome of Tat/Grb2 interaction. *Biochim Biophys Acta* **1813**, 1836-1844 (2011). https://doi.org:10.1016/j.bbamcr.2011.06.012

6 Garrido-Rodríguez, V. *et al.* Dysregulation of iron metabolism modulators in virologically suppressed HIV-infected patients. *Front Immunol* **13**, 977316 (2022). https://doi.org:10.3389/fimmu.2022.977316

7 Reis, M. L., Maeda, S., Rosatelli, J. B., Donadi, E. A. & Roselino, A. M. Kininogens and kallikrein in pruritic papular eruption. *Immunopharmacology* **45**, 115-120 (1999). https://doi.org:10.1016/s0162-3109(99)00063-6

8 Congote, L. F. Monitoring insulin-like growth factors in HIV infection and AIDS. *Clin Chim Acta* **361**, 30-53 (2005). https://doi.org:10.1016/j.cccn.2005.05.001

9 Kessler, M. *et al.* Growth patterns in pubertal HIV-infected adolescents and their correlation with cytokines, IGF-1, IGFBP-1, and IGFBP-3. *J Pediatr Endocrinol Metab* **26**, 639-644 (2013). https://doi.org:10.1515/jpem-2011-0464

10 Schaffrath, R. & Brinkmann, U. Diphthamide - a conserved modification of eEF2 with clinical relevance. *Trends Mol Med* (2023). https://doi.org:10.1016/j.molmed.2023.11.008

11 Wang, Y., Branicky, R., Noë, A. & Hekimi, S. Superoxide dismutases: Dual roles in controlling ROS damage and regulating ROS signaling. *J Cell Biol* **217**, 1915-1928 (2018). https://doi.org:10.1083/jcb.201708007

12 Aukrust, P. *et al.* Disturbed glutathione metabolism and decreased antioxidant levels in human immunodeficiency virus-infected patients during highly active antiretroviral therapy--potential immunomodulatory effects of antioxidants. *J Infect Dis* **188**, 232-238 (2003). https://doi.org:10.1086/376459

13 Pugliese, C. *et al.* Assessment of antioxidants status and superoxide dismutase activity in HIV-infected children. *Braz J Infect Dis* **18**, 481-486 (2014). https://doi.org:10.1016/j.bjid.2014.02.003

14 Lopes, G. O. *et al.* Increased vascular function and superoxide dismutase activity in physically active vs inactive adults living with HIV. *Scand J Med Sci Sports* **29**, 25-33 (2019). https://doi.org:10.1111/sms.13312

15 Wignot, T. M., Stewart, R. P., Schray, K. J., Das, S. & Sipos, T. In vitro studies of the effects of HAART drugs and excipients on activity of digestive enzymes. *Pharm Res* **21**, 420-427 (2004). https://doi.org:10.1023/B:PHAM.0000019294.03188.cf

16 Yousefi, F. & Roozbeh, F. The effect of coenzyme Q10 in comparison with placebo on CD4 in HIV-infected patients. *Clinical Epidemiology and Global Health* **7**, 306-308 (2019). https://doi.org:https://doi.org/10.1016/j.cegh.2018.10.001

17 Williams, K. S., Killebrew, D. A., Clary, G. P. & Meeker, R. B. Opposing Effects of NGF and proNGF on HIV Induced Macrophage Activation. *J Neuroimmune Pharmacol* **11**, 98-120 (2016). https://doi.org:10.1007/s11481-015-9631-z

18 Deletsu, S. D. *et al.* Identification and characterization of Stathmin 1 as a host factor involved in HIV-1 latency. *Biochem Biophys Res Commun* **567**, 106-111 (2021). https://doi.org:10.1016/j.bbrc.2021.06.017
19 Teer, E., Joseph, D. E., Dominick, L., Glashoff, R. H. & Essop, M. F. Expansion of GARP-Expressing CD4(+)CD25(-)FoxP3(+) T Cells and SATB1 Association with Activation and Coagulation in Immune Compromised HIV-1-Infected Individuals in South Africa. *Virol Sin* **36**, 1133-1143 (2021). https://doi.org:10.1007/s12250-021-00386-8
20 Yuferov, V. *et al.* Expression of ephrin receptors and ligands in postmortem brains of HIV-infected subjects with and without cognitive impairment. *J Neuroimmune Pharmacol* **8**, 333-344 (2013). https://doi.org:10.1007/s11481-012-9429-1
21 Tuckermann, J. P., Pittois, K., Partridge, N. C., Merregaert, J. & Angel, P. Collagenase-3 (MMP-13) and integral membrane protein 2a (Itm2a) are marker genes of chondrogenic/osteoblastic cells in bone formation: sequential temporal, and spatial expression of Itm2a, alkaline phosphatase, MMP-13, and osteocalcin in the mouse. *J Bone Miner Res* **15**, 1257-1265 (2000). https://doi.org:10.1359/jbmr.2000.15.7.1257
22 Van den Plas, D. & Merregaert, J. Constitutive overexpression of the integral membrane protein Itm2A enhances myogenic differentiation of C2C12 cells. *Cell Biol Int* **28**, 199-207 (2004). https://doi.org:10.1016/j.cellbi.2003.11.019
23 Dreier, R., Opolka, A., Grifka, J., Bruckner, P. & Grässel, S. Collagen IX-deficiency seriously compromises growth cartilage development in mice. *Matrix Biol* **27**, 319-329 (2008). https://doi.org:10.1016/j.matbio.2008.01.006
24 Wang, Y. *et al.* IGF-1R signaling in chondrocytes modulates growth plate development by interacting with the PTHrP/Ihh pathway. *J Bone Miner Res* **26**, 1437-1446 (2011). https://doi.org:10.1002/jbmr.359
25 Wu, S., Yang, W. & De Luca, F. Insulin-Like Growth Factor-Independent Effects of Growth Hormone on Growth Plate Chondrogenesis and Longitudinal Bone Growth. *Endocrinology* **156**, 2541-2551 (2015). https://doi.org:10.1210/en.2014-1983
26 Hawkes, C. P. & Grimberg, A. Insulin-Like Growth Factor-I is a Marker for the Nutritional State. *Pediatr Endocrinol Rev* **13**, 499-511 (2015).
27 Lee, S. E. *et al.* The Plasma Proteome Is Associated with Anthropometric Status of Undernourished Nepalese School-Aged Children. *J Nutr* **147**, 304-313 (2017). https://doi.org:10.3945/jn.116.243014
28 Li, Q., Pu, D., Xia, X., Liu, H. & Li, L. Serum Concentrations of Cartilage Intermediate Layer Protein 2 Were Higher in Overweight and Obese Subjects. *Biomed Res Int* **2022**, 6290064 (2022). https://doi.org:10.1155/2022/6290064
29 Xiong, S. Q. *et al.* Role of endogenous insulin gene enhancer protein ISL-1 in angiogenesis. *Mol Vis* **22**, 1375-1386 (2016).
30 Iruela-Arispe, M. L., Liska, D. J., Sage, E. H. & Bornstein, P. Differential expression of thrombospondin 1, 2, and 3 during murine development. *Dev Dyn* **197**, 40-56 (1993). https://doi.org:10.1002/aja.1001970105
31 Hankenson, K. D., Hormuzdi, S. G., Meganck, J. A. & Bornstein, P. Mice with a Disruption of the Thrombospondin 3 Gene Differ in Geometric and Biomechanical Properties of Bone and Have Accelerated Development of the Femoral Head. *Molecular and Cellular Biology* **25**, 5599-5606 (2005). https://doi.org:10.1128/MCB.25.13.5599-5606.2005
32 Niu, N., Shao, R., Yan, G. & Zou, W. Bromodomain and Extra-terminal (BET) Protein Inhibitors Suppress Chondrocyte Differentiation and Restrain Bone Growth. *J Biol Chem* **291**, 26647-26657 (2016). https://doi.org:10.1074/jbc.M116.749697
33 Mudibo, E. O. *Systemic biological mechanisms underpin poor post-discharge growth among severely wasted children with HIV: HIV-SM-PROTEOMICS*, <https://doi.org/10.5281/zenodo.14060281> (2024).
