# Supplementary material for: Systemic biological mechanisms underpin poor post-discharge growth among severely wasted children with HIV
Source: Nat Commun. 2024 Nov 27;15:10299. doi: 10.1038/s41467-024-54717-w (PMC11603168; doi:10.1038/s41467-024-54717-w)
Supplement: Supplementary file 3 — Description of Additional Supplementary Files [file 41467_2024_54717_MOESM3_ESM.pdf]

Description of Additional Supplementary Files

File Name: Supplementary Data 1
Description: An Excel file of Supplementary Data containing structural equation model parameter estimates and fit measures
