# Supplementary material for: Systemic biological mechanisms underpin poor post-discharge growth among severely wasted children with HIV
Source: Nat Commun. 2024 Nov 27;15:10299. doi: 10.1038/s41467-024-54717-w (PMC11603168; doi:10.1038/s41467-024-54717-w)
Supplement: Supplementary file 5 — Reporting Summary [file 41467_2024_54717_MOESM5_ESM.pdf]

Corresponding author(s): Evans O. Mudibo
James M. Njunge
Gerard Bryan Gonzales

Last updated by author(s): November 13 2024

Reporting Summary

Nature Portfolio wishes to improve the reproducibility of the work that we publish. This form provides structure for consistency and transparency in reporting. For further information on Nature Portfolio policies, see our Editorial Policies and the Editorial Policy Checklist.

Statistics

For all statistical analyses, confirm that the following items are present in the figure legend, table legend, main text, or Methods section.

n/a	Confirmed	
☐	☒	The exact sample size (*n*) for each experimental group/condition, given as a discrete number and unit of measurement
☐	☒	A statement on whether measurements were taken from distinct samples or whether the same sample was measured repeatedly
☐	☒	The statistical test(s) used AND whether they are one- or two-sided<br>*Only common tests should be described solely by name; describe more complex techniques in the Methods section.*
☐	☒	A description of all covariates tested
☐	☒	A description of any assumptions or corrections, such as tests of normality and adjustment for multiple comparisons
☐	☒	A full description of the statistical parameters including central tendency (e.g. means) or other basic estimates (e.g. regression coefficient) AND variation (e.g. standard deviation) or associated estimates of uncertainty (e.g. confidence intervals)
☐	☒	For null hypothesis testing, the test statistic (e.g. *F*, *t*, *r*) with confidence intervals, effect sizes, degrees of freedom and *P* value noted<br>*Give P values as exact values whenever suitable.*
☒	☐	For Bayesian analysis, information on the choice of priors and Markov chain Monte Carlo settings
☐	☒	For hierarchical and complex designs, identification of the appropriate level for tests and full reporting of outcomes
☒	☐	Estimates of effect sizes (e.g. Cohen's *d*, Pearson's *r*), indicating how they were calculated

Our web collection on statistics for biologists contains articles on many of the points above.

Software and code

Policy information about availability of computer code

Data collection

The current study is a retrospective analysis nested within a larger cohort study. The current study used available datasets that included clinical data such as clinical syndromes (malaria, diarrhoea, pneumonia, TB, HIV status); demographic data, sex, age, site of enrollment; and plasma proteomics data quantified using the SomaScan Assay by SomaLogic Inc. Therefore, for this analysis, no data collection was done.

Data analysis

All the analyses reported in this study were done using R version 4.3.1 in RStudio 2023.06.1+524.pro1. The codes used in this study are hosted on the Harvard Dataverse website under DOI: https://doi.org/10.7910/DVN/D8HZLJ. Similar versions of the codes are deposited on the corresponding author's GitHub repository at https://github.com/mudiboevans/HIV-SM-PROTEOMICS. This GitHub repository has been linked to Zenodo under DOI: https://doi.org/10.5281/zenodo.14060281. Below are the packages and respective versions:

```
library(SomaDataIO) # version 6.0.0
library(WGCNA) # version 1.73
library(gridExtra) # version 2.3
library(CorLevelPlot) # version
library(dplyr) # version 1.1.4
library(tidyr) # version 1.3.1
library(readr) # version 2.1.5
library(readxl) # version 1.4.3
library(ggplot2) # version 3.5.1
library(vroom) # version 1.6.5
library(janitor) # version 2.2.0
library(ggpubr) # version 0.6.0
```

```
library(cowplot) # version 1.1.3
library(tidyverse) # version 2.0.0
library(ggsci) # version 3.2.0
library(gtsummary) # version 2.0.3
library(gt) # version 0.11.1
library(webshot2) # version
library(webshot) # version 0.5.5
library(plm) # version 2.6-4
library(rstatix) # version 0.7.2
libray(lmerTest) # version 3.1-3
library(lme4) # version 1.1-35.5
library(VGAM) # version 1.1-12
library(kableExtra) # version 1.4.0
library(pbkrtest) # verson 0.5.3
library(lavaan) # version 0.6-19
library(lavaanPlot) # version 0.8.1
```

For manuscripts utilizing custom algorithms or software that are central to the research but not yet described in published literature, software must be made available to editors and reviewers. We strongly encourage code deposition in a community repository (e.g. GitHub). See the Nature Portfolio guidelines for submitting code & software for further information.

Data

Policy information about availability of data

All manuscripts must include a data availability statement. This statement should provide the following information, where applicable:

- Accession codes, unique identifiers, or web links for publicly available datasets
- A description of any restrictions on data availability
- For clinical datasets or third party data, please ensure that the statement adheres to our policy

The data supporting findings of this study is available at Havard Dataverse repository through this link https://doi.org/10.7910/DVN/D8HZLJ. The data contain sensitive information about study participants and may include identifiers that could compromise confidentiality or lead to ethnic stigmatisation. To ensure participant privacy and compliance with ethical guidelines and data protection regulations, access to the data is restricted. Access to the data require submission of a formal request to the data governance committee via dgc@kemri-wellcome.org. The requester provides investigators details, variables requested, intended use of the dataset, potential risks of the study including risks to confidentiality of individuals or communities, potential benefits of the study including to participant communities, scientific capacity building or health policy and planned outputs (if analysis on dataset will result in publication or reports or presentations). The requester also needs to formally agree to the conditions and limitations for data sharing to avoid misuse of shared data. Details on how to request the data and submission of a data request form are found on the Havard Dataverse website using this link https://doi.org/10.7910/DVN/D8HZLJ.

Research involving human participants, their data, or biological material

Policy information about studies with human participants or human data. See also policy information about sex, gender (identity/presentation), and sexual orientation and race, ethnicity and racism.

Reporting on sex and gender	In this study sex was defined based on the biological attribute. Therefore, male and female as used in the study, implied the sex of the participants. Based on sex, the proportion of children with and without HIV was comparable. No sex stratified analyses were performed. However, cognizant of potential effect of sex on growth and biological processes, we adjusted for sex in all our models. The median age of children included in the study was 12.6 months.
Reporting on race, ethnicity, or other socially relevant groupings	This study did not group the participants based on their races, ethnicity or any social groups. The current study included participants recruited at 6 different sites (hospitals) from 4 sub-Saharan Africa countries. Because of the potential site-specific effects, site was included in the regression models as a random effect.
Population characteristics	The study focused on children with and without HIV. Cases were children with HIV while controls included those without HIV. The median age in months between these groups was comparable. At discharge, children with HIV were more wasted, underweight and stunted than children without HIV. Irrespective of the HIV status, common clinical symptoms at admission to hospital included diarrhea, pneumonia and lower respiratory tract infection.
Recruitment	This is a case-control study nested within the Childhood Acute Illness and Nutrition Network cohort in sub-Saharan Africa. Cases were all children with HIV alive at hospital discharge while controls comprised of randomly selected HIV negative children. To balance the baseline characteristics between children with and without HIV we implemented inverse probability weighting.
Ethics oversight	The study protocol was reviewed and approved by the Oxford Tropical Research Ethics Committee, United Kingdom; Scientific and Ethical Review Unit (SERU), Kenya Medical Research Institute, Kenya; Makerere University School of Biomedical Sciences Research Ethics Committee and The Uganda National Council for Science and Technology, Uganda; The University of Malawi and COMREC, Kamuzu University of Health Sciences, Malawi; The University of Ouagadougou and Comité d'éthique institutionnel du Centre MURAZ, Burkina Faso.

Note that full information on the approval of the study protocol must also be provided in the manuscript.

Field-specific reporting

Please select the one below that is the best fit for your research. If you are not sure, read the appropriate sections before making your selection.

☒ Life sciences ☐ Behavioural & social sciences ☐ Ecological, evolutionary & environmental sciences

For a reference copy of the document with all sections, see nature.com/documents/nr-reporting-summary-flat.pdf

Life sciences study design

All studies must disclose on these points even when the disclosure is negative.

Sample size

No formal sample size calculation was performed for this secondary analysis study. The study included all children with HIV (cases) that met the inclusion criteria of the study. For the comparative analysis a total of 834 children were analyzed (HIV+; n = 112) meaning for this part of the analysis, for every case at least seven controls were randomly selected. To understand the systemic proteome signatures associated with HIV status the study analysed 689 children of whom 79 had HIV. For mechanistic analysis that focused on the effect of HIV on post-discharge growth of children we only focused on severely wasted children n = 217 of whom 38 were HIV positive. These children were drawn from a nested case-cohort study, a random selection from the entire population.

Data exclusions

The research questions guided which participants were excluded for each analysis. To understand growth trajectories and effect of HIV on six months post-discharge growth, the study excluded children who died during the study. To examine the relationship between HIV status and protein modules we further excluded any child who lacked proteome data. Finally, to determine the impact of HIV-associated protein modules on post-discharge growth among severely wasted, we only focused on the severely wasted children at discharge because these are group of children with greatest growth deficits at baseline and are therefore expected to have greater anthropometric gains during convalescence.

Replication

The results can be reproduced since data and codes are available but will await validation in future studies. In addition, our structural equation models were reviewed by an independent collaborator.

Randomization

This was a case-control study and HIV status was used to classify the study participants.

Blinding

This being a retrospective analysis of a data, blinding of the groups was not applicable.

Reporting for specific materials, systems and methods

We require information from authors about some types of materials, experimental systems and methods used in many studies. Here, indicate whether each material, system or method listed is relevant to your study. If you are not sure if a list item applies to your research, read the appropriate section before selecting a response.

Materials & experimental systems

n/a	Involved in the study	
☒	☐	Antibodies
☒	☐	Eukaryotic cell lines
☒	☐	Palaeontology and archaeology
☒	☐	Animals and other organisms
☒	☐	Clinical data
☒	☐	Dual use research of concern
☒	☐	Plants

Methods

n/a	Involved in the study	
☒	☐	ChIP-seq
☒	☐	Flow cytometry
☒	☐	MRI-based neuroimaging

Plants

Seed stocks

Report on the source of all seed stocks or other plant material used. If applicable, state the seed stock centre and catalogue number. If plant specimens were collected from the field, describe the collection location, date and sampling procedures.

Novel plant genotypes

Describe the methods by which all novel plant genotypes were produced. This includes those generated by transgenic approaches, gene editing, chemical/radiation-based mutagenesis and hybridization. For transgenic lines, describe the transformation method, the number of independent lines analyzed and the generation upon which experiments were performed. For gene-edited lines, describe the editor used, the endogenous sequence targeted for editing, the targeting guide RNA sequence (if applicable) and how the editor was applied.

Authentication

Describe any authentication procedures for each seed stock used or novel genotype generated. Describe any experiments used to assess the effect of a mutation and, where applicable, how potential secondary effects (e.g. second site T-DNA insertions, mosiacism, off-target gene editing) were examined.
